# Role of computed tomography angiography on the management of overt obscure gastrointestinal bleeding

**DOI:** 10.1371/journal.pone.0172754

**Published:** 2017-03-01

**Authors:** Chao-Ming Tseng, I-Chang Lin, Chi-Yang Chang, Hsiu-Po Wang, Chih-Cheng Chen, Lein-Ray Mo, Jaw-Town Lin, Chi-Ming Tai

**Affiliations:** 1 Department of Internal Medicine, E-Da Hospital, I-Shou University, Kaohsiung City, Taiwan; 2 Taiwan Association for the Study of Small Intestinal Diseases, Taoyuan city, Taiwan; 3 Department of Radiology, E-Da Hospital, I-Shou University, Kaohsiung City, Taiwan; 4 Department of Internal Medicine, National Taiwan University Hospital, National Taiwan University, Taipei, Taiwan; 5 Division of Gastroenterology, Tainan Municipal Hospital, Tainan City, Taiwan; 6 School of Medicine and Big Data Research Centre, Fu Jen Catholic University, New Taipei City, Taiwan; University Hospital Llandough, UNITED KINGDOM

## Abstract

**Background and aim:**

The role of computed tomography angiography (CTA) on the management of acute overt obscure gastrointestinal bleeding (OGIB) remains unclear. We designed a study to evaluate the impact of CTA before enteroscopy for acute overt OGIB.

**Methods:**

All patients undergoing CTA followed by enteroscopy for acute overt OGIB were enrolled in this retrospective study. Clinical characteristics and diagnosis were compared between patients with positive and negative CTA findings. We evaluated the impact of CTA on subsequent enteroscopy.

**Results:**

From February 2008 to March 2015, 71 patients including 25 patients with positive CTA findings and 46 patients with negative CTA findings, were enrolled. All 25 patients with positive CTA findings were confirmed to have mid GI lesions, a significantly higher proportion than among patients with negative CTA findings (100% vs. 52.2%, respectively; *P* <0.001). CTA had a higher diagnostic yield for bleeding from tumor origin than from non-tumor origin (80.0% vs. 23.7%, respectively; *P* <0.001). The diagnostic yield of CTA and enteroscopy was 35.2% and 73.2%, respectively. The lesions could be identified by the initial route of enteroscopy in more patients with positive CTA findings than in those with negative CTA findings (92.0% vs. 47.8%, respectively; *P* <0.001). Lesions could be identified in seven of the 25 patients (28.0%) with positive CTA findings by using only push enteroscopy instead of single-balloon enteroscopy (SBE), but all 46 patients with negative CTA findings needed SBE for deep small-bowel examination.

**Conclusions:**

CTA is useful in the diagnosis of acute overt OGIB, especially in patients with bleeding from tumors. In addition, it also can show the precise location of bleeding, and guide subsequent enteroscopic management.

## Introduction

Obscure gastrointestinal bleeding (OGIB), which accounts for 5% of patients presenting with GI hemorrhage, is defined as bleeding from the gastrointestinal (GI) tract that persists or recurs after routine endoscopic studies [1, 2]. Up to 75% of patients with OGIB have small bowel lesions [1]. OGIB can be classified as overt or occult according to the presence or absence of clinically evident bleeding [1]. The diagnosis of the cause of the overt OGIB remains a clinical challenge, and leads to extensive and repetitive examinations. Capsule endoscopy (CE) is recommended as the first diagnostic tool for OGIB [1, 3, 4], and the results of CE can guide subsequent deep enteroscopy; however, CE has several limitations, including failure to detect extra-luminal lesions, visualization limited by blood, capsule retention, and high cost [5, 6].

Computed tomography angiography (CTA) is a rapid and noninvasive examination that allows visualization of the site of active bleeding which is seen as focal contrast extravasation or an extra-luminal tumor [1, 6, 7]. Although CTA is reported to be a good diagnostic tool in patients with acute GI bleeding throughout the GI tract [8], studies reporting the role of CTA in the management of overt OGIB are scarce. Yen et al. reported that CTA is a useful triage tool in patients with overt OGIB, but the number of cases was small and only half the patients with OGIB had undergone CTA before enteroscopic examination [6]. In recently reported guidelines, others have strongly recommended CTA for the management of overt OGIB in patients with active bleeding, but the level of evidence was low [9]. In order to fill in missing data, we performed this study to evaluate the clinical role of CTA in patients with active overt OGIB.

## Materials and methods

### Eligible patients

Overt OGIB is defined as melena or hematochezia after a non-diagnostic upper and lower endoscopic examination. CTA has been the primary diagnostic tool for the management of patients with overt OGIB in E-Da Hospital, Kaohsiung, Taiwan, and has been performed within 24 hours in all patients with acute overt OGIB before enteroscopy except for those with contraindications to CTA, such as renal insufficiency or allergy to contrast medium. The choice of the insertion route and types of subsequent enteroscopy are dependent on the results of CTA. Intra-operative enteroscopy is performed if SBE fails to detect the source of persistent bleeding. All patients with acute overt OGIB who underwent CTA followed by enteroscopy were enrolled in this retrospective study. Demographic data, medication history, comorbidities, and final diagnosis were recorded. Chronic renal failure was defined as plasma creatinine more than 2.0 mg/dL. Patients receiving regular dialysis for chronic renal failure were defined as the patients with end stage renal disease (ESRD). If the sources of bleeding were detected, these would be classified as upper, lower, or mid GI lesions, according to the location. Upper GI lesions referred to the bleeding sites proximal to the papilla of Vater. Lower GI lesions consisted of those bleeding from the colon and rectum. Mid GI lesions referred to small bowel bleeding, between the papilla of Vater and terminal ileum [10]. The study was approved by the Ethical Committee of E-Da Hospital. The Ethics Committee waived the need for consent, and all the data were de-identified prior to analysis.

### Computed tomography procedure

We performed CTA on all the patients before a procedure with a multidetector computed tomography scanner (Sensation 16, SIEMENS Corp, Washington, DC). The study included precontrast and contrast-enhanced triphasic scans of the abdomen in a craniocaudal direction. No oral contrast material was given before the examination. A total dose of 100 mL of iodinated contrast was injected through a peripheral vein, followed by 40 mL normal saline, with an injection rate of 3 mL/sec. For triphasic acquisitions, scanning was started with a 10-sec scan delay for the hepatic arterial phase after the attenuation value of the aorta reached 100 HU. Twenty-five seconds after the end point of the hepatic arterial phase, the scans for the portal venous phase were acquired. Late-phase images were acquired 20 sec after the end of the acquisition of the portal venous phase.

#### Diagnostic performance of CT angiography

The finding of extravasation of contrast in the bowel lumen is considered to be diagnostic of active gastrointestinal bleeding. The detection of a small bowel mass, thickened bowel wall, or bowel vascular abnormalities is also considered to represent positive CTA findings. The diagnostic outcomes of CTA were as follows: (1) false-negative: negative CTA result, with the source of bleeding detected by subsequent small bowel examinations or persistent or intermittent bleeding; (2) true-negative: verification of negative CT angiography by subsequent negative small bowel examinations and no bleeding episodes noted during a follow-up period of at least 6 months; (3) true-positive: confirmation of CTA finding by subsequent small bowel examinations, such as enteroscopy or surgery [11].

### Enteroscopic procedures

Push enteroscopy (Olympus SIF-Q260, Olympus Medical Systems Corp) or single-balloon enteroscopy (SBE) was performed for the management of acute overt OGIB. SBE was performed by using the Olympus SIF-Q260 enteroscope and disposable overtube (ST-SB1, Olympus Medical Systems Corp). All enteroscopic procedures were performed by two experienced endoscopists (CM Tseng and CM Tai). If the CTA showed that the potential source of bleeding was in the distal duodenum or proximal jejunum, push enteroscopy via the oral route was performed first. Otherwise, we used SBE for deep small-bowel examination.

The choice of route of insertion for SBE was also dependent on the results of CTA. If the CTA identified a lesion that was a plausible source of bleeding, we chose the route of insertion accordingly. If CTA was negative, the insertion route depended on the color of the stool: the oral route for melena and the anal route for hematochezia. If SBE failed to detect the source of bleeding from one route, tattooing was performed at the deepest point, and an additional session of SBE was performed from the other route.

### Statistical analysis

Results described as continuous variables were presented as the mean and standard deviation, and those described as categorical variables were given as percentages. Comparisons between patients with positive CTA findings and patients with negative CTA findings were assessed using Student's t-test, chi-square test, or Fisher's exact test when appropriate. A *P*-value <0.05 indicated a statistically significant difference. All analyses were performed by SPSS 22.0 version for Windows (IBM Corp, Armonk, NY).

## Results

### Patient characteristics

**Fig 1:**
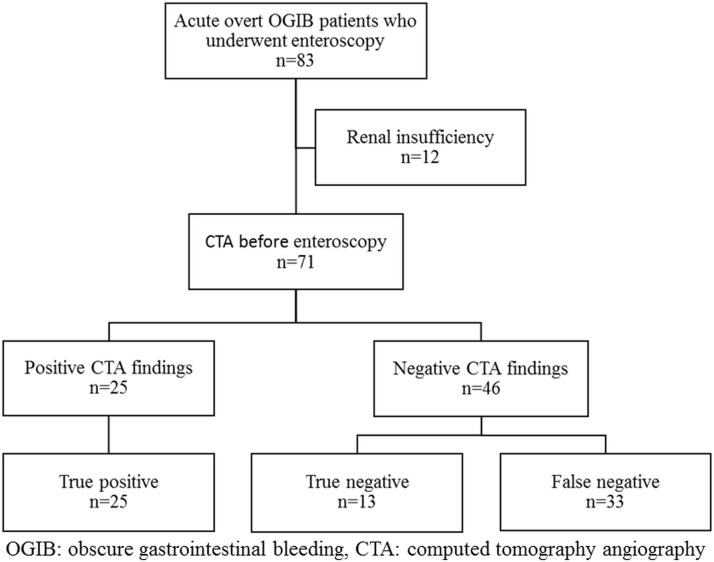
Flowchart showing recruitment for the study participants.

Between February 2008 and March 2015, a total of 71 patients with acute overt OGIB underwent CTA followed by enteroscopy at E-Da Hospital. Three patients with OGIB and positive CTA findings were excluded because they had not received enteroscopic examinations. Two patients received angiographic embolization directly due to colonic diverticular bleeding and unstable hemodynamic, and one patient received surgical resection directly for gastrointestinal stromal tumor (GIST) bleeding. Twelve patients did not undergo CTA due to chronic renal failure. A total of 71 patients (36 men [50.7%] and 35 women [49.3%]) with a mean age of 62.4 ±15.1 years were enrolled in the present study (Fig 1). A bleeding source could be identified by enteroscopy or a surgical procedure in 58 of the 71 patients (81.7%). Mid, upper, and lower GI lesions were found in 49 (69.0%), 2 (2.8%), and 7 (10.0%) patients, respectively. Tumor (28.2%) was the most common mid GI lesion, followed by angioectasia (12.7%), ulcer (12.7%), diverticulum (8.5%), Dieulafoy’s lesion (5.6%), and varix (1.4%) (Table 1). Twenty-five patients were diagnosed by operation: 20 patients due to small bowel tumor, 4 patients due to persistent bleeding despite of negative findings of SBE and CTA, and one patient due to failure of SBE to reach the bleeder identified by CTA. The other 33 patients were diagnosed by the endoscopic examination only. Thirteen patients had no specific diagnosis after SBE and abdominal CTA examination, and no rebleeding episodes were detected during the follow-up period, for up to 6 months.

### Findings of CTA and enteroscopy

Table 1 shows a comparison of clinical characteristics and final diagnosis between patients with positive CTA findings and those with negative CTA findings. There was no significant difference in clinical characteristics between the two groups. All 25 patients with positive CTA findings were confirmed to have midGI lesions, which was significantly higher than the proportion of patients with negative CTA findings who had mid GI lesions (100% vs. 52.2%, respectively; *P* <0.001). The CTA features of the 25 patients with positive CTA findings were small bowel tumor in 15 patients, extravasation in five patients, thickened bowel wall in 3 patients, and vascular lesions in 2 patients. Sixteen of the 20 patients (80%) with confirmed diagnosis of tumors as the cause of overt OGIB were identified by CTA, 15 as small bowel tumors and one as thickened bowel wall. Of note, 4 small or flat tumors were not detected by CTA, including GIST, lymphoma, lymphangiomatosis, and metastatic tumor (angiosarcoma). More patients with tumors were found in the group with positive CTA findings than in the group with negative CTA findings (64% vs. 8.7%, respectively; *P* <0.001).

**Table 1:** Comparison of clinical characteristics and final diagnosis between patients with positive CTA findings and patients with negative CTA finding.

	All	Positive CTA	Negative CTA	*P* ^*a*^
	n = 71	n = 25	n = 46	
**Age in years, mean (SD)**	62.4 (15.1)	60.9 (11.0)	63.1 (16.9)	0.513
**Male sex, n (%)**	36 (50.7)	11 (44.0)	25 (54.3)	0.405
**Underlying disease, n (%)**				
DM	15 (21.1)	2 (8.0)	13 (28.3)	0.067
Cirrhosis	12 (16.9)	2 (8.0)	10 (21.7)	0.193
ESRD	11 (15.5)	3 (12.0)	8 (17.4)	0.736
Drug^b^	8 (11.3)	2 (8.0)	5 (10.9)	1
Initial Hb in g/dl, mean (SD)	7.3 (2.0)	7.2 (2.4)	7.4 (1.8)	0.646
Transfusion	64 (90.1)	23 (92.0)	41 (89.1)	1
**Mid GI lesion, n (%)**	49 (69.0)	25 (100.0)	24 (52.2)	<0.001
***Tumor***	20 (28.2)	16 (64.0)	4 (8.7)	<0.001
GIST	11 (15.5)	10 (40)	1 (2.2)	
Lymphoma	2 (2.8)	1 (4.0)	1 (2.2)	
Adenocarcinoma	1 (1.4)	1 (4.0)	0 (0)	
Metastasis	4 (5.6)	3 (12.0)	1 (2.2)	
Paraganglioma	1 (1.4)	1 (4.0)	0 (0)	
Lymphangiomatosis	1 (1.4)	0 (0)	1 (2.2)	
***Non-tumor***	29 (40.8)	9 (36.0)	20 (43.5)	0.345
Diverticulum	6 (8.5)	1 (4.0)	5 (10.9)	
Angioectasia	9 (12.7)	2 (8.0)	7 (15.2)	
Ulcer	9 (12.7)	3 (12.0)	6 (13.0)	
Varix	1 (1.4)	1 (4.0)	0 (0)	
Dieulafoy lesion	4 (5.6)	2 (8.0)	2 (4.3)	
**Upper GI lesion**	2 (2.8)	0 (0)	2 (4.3)	
**Lower GI lesion**	7 (10.0)	0 (0)	7 (15.2)	
**Undiagnosed**	13 (18.3)	0 (0)	13 (28.3)	

a: Positive CTA v.s negative CTA

b: Non-steroidal anti-inflammatory agents /anticoagulants/antiplatelet agents

CTA: computed tomography angiography, DM: diabetes mellitus, ESRD: end-stage renal disease, Hb: hemoglobin, GI: gastrointestinal, GIST: Gastrointestinal stromal tumor

Comparisons between patients with positive CTA findings and patients with negative CTA findings were assessed using Student's t-test, chi-square test, or Fisher's exact test when appropriate.

### Diagnostic yield of CTA and enteroscopy

All positive CTA findings (n = 25) were confirmed by enteroscopy or surgical exploration, and therefore were regarded as true positives. Among the 46 patients with negative CTA findings, 33 (71.7%) of the findings were regarded as false negative, and 13 (28.3%) were regarded as true negative (Fig 1). Among the 58 patients with the source of bleeding identified, 52 patients had the source identified by enteroscopy. Of the 6 cases in which enteroscopy failed to detect the source of bleeding, 3 were diagnosed by intraoperative enteroscopy. Two cases presenting with rebleeding and shock were diagnosed by subsequent conventional angiography. One case was diagnosed by subsequent colonoscopy as diverticular bleeding. The diagnostic yield of CTA and enteroscopy was 35.2% and 73.2%, respectively. The sensitivity, specificity, positive predictive value, and negative predictive value of CTA for overt OGIB were: 43.1%, 100%, 100%, and 28.3%, respectively.

### Clinical impact of CTA

Table 2 shows the clinical impact of CTA on the subsequent choice of insertion route and type of enteroscopy. The sources of bleeding could be identified by the initial route of enteroscopy in more patients with positive CTA findings than with negative CTA findings (92.0% vs. 47.8%, *P* <0.001). Two patients with positive CTA findings needed a second SBE via a different route. One patient was found on CTA to have contrast extravasation from the middle part of the small intestine, which could not be reached by the initial SBE. The other patient was found on CTA to have one 8-cm mass in the lower abdomen. In this patient, we initially chose to perform the SBE via the anal route; however, this patient was ultimately diagnosed with a large GIST originating in the jejunum. In addition, seven of the 25 patients (28.0%) with positive CTA findings were found to have distal duodenum or proximal jejunum lesions by using only push enteroscopy instead of SBE. However, all 46 patients with negative CTA findings needed SBE for deep small-bowel examination

**Table 2:** Clinical impact of CTA on choice of insertion route and type of enteroscopy.

	Positive CTA	Negative CTA	*P* value
	n = 25	n = 46	
**Route choice**			
Correct, n (%)	23 (92.0)	22 (47.8)	<0.001
Incorrect, n (%)	2 (8.0)	24 (52.2)	
**Enteroscopy**			
Push, n (%)	7 (28.0)	0 (0)	<0.001
SBE, n (%)	18 (72.0)	46 (100)	

CTA: computed tomography angiography, SBE: single-balloon enteroscopy.

Comparisons between patients with positive CTA findings and patients with negative CTA findings were assessed using chi-square test, or Fisher's exact test when appropriate.

## Discussion

Our study demonstrated the efficacy of CTA before enteroscopy in the management of patients with acute overt OGIB. The diagnostic yield of CTA was 35.2%; in addition, CTA may aid in choosing an easier and more efficient method of enteroscopy. CTA may also help to identify the preferable insertion route in 92.0% of patients with positive CTA findings. If CTA demonstrates the site of bleeding in the distal duodenum or proximal jejunum, the endoscopist may use push enteroscopy instead of deep enteroscopy.

A decision analysis revealed that an initial approach of deep enteroscopy is cost-effective, and can provide timely treatment for patients with OGIB [12]; however, deep enteroscopy is an intrinsically invasive and labor-intensive procedure with a pooled major complication rate of 0.72% [13, 14]. It is helpful to know the location of the source of bleeding before performing deep enteroscopy. In our studies, in patients with positive CTA, the choice of initial route of SBE was more likely to be correct, compared with patients with negative CTA. Positive CTA can guide the subsequent SBE efficiently by choosing the preferable insertion route and type of enteroscopy. Without a pre-enteroscopic imaging study, the choice of insertion route of SBE usually depends on clinical symptoms, such as stool color, which may lead to a suboptimal route of insertion. Robles et al. demonstrated that capsule endoscopy before emergent double-balloon enteroscopy could correctly inform the initial route of enteroscopy compared with that based only on clinical data in 25% of cases [15].

The diagnostic yield of CTA was comparable to that described in previous studies [1, 6, 7]. Although only 5 of 58 patients (8.6%) with the source of bleeding identified were found to have extravasation on CTA, other features such as small bowel tumor, thickened bowel wall, or vascular lesions on CTA also could provide useful information regarding the location of the source of bleeding. We were able to diagnose an additional 20 of 58 patients (34.5%) according to these features.

Computed tomography has been reported to have a high diagnostic yield for small bowel tumors [5, 16, 17]. In our study, the diagnostic yield of CTA for small bowel neoplasms was 80%, which is similar to that described in previous studies [5, 16, 17]; however, despite a good diagnostic yield, 4 cases with small or flat tumors (20%) were not detected by CTA. The ability of CTA to detect non-tumor lesions is relatively limited. In our study, the diagnostic yield of CTA for the non-tumor lesion was only 31% (9/29). Among the 24 patients with negative CTA findings whose source of bleeding was diagnosed by enteroscopy, 14 (58.3%) had vascular lesions. False-negative results of CTA may occur due to intermittent bleeding of a vascular lesion [18]. CT enterography uses a large volume of oral contrast to distend the bowel lumen and facilitate detection of mucosal lesions [19, 20]. In a meta-analysis, CT enterography was reported to have a diagnostic yield of 40% in patients with OGIB [21]; however, it was not feasible for patients with acute overt OGIB because it may delay subsequent enteroscopic examination [6].

We found that the most common mid GI lesion in 83 patients with acute overt OGIB was tumor (24.1%), followed by angioectasia (16.9%) and ulcer (14.5%). Consistent with our results, Zhang et al. also reported that bleeding from small bowel tumors was the most common finding in Chinese patients with massive OGIB [22]. In contrast, several studies found that the most common etiology of OGIB was angioectasia [23–25]. This discrepancy was probably caused by selection bias. We enrolled only patients with acute overt OGIB; studies enrolling both patients with occult and patients with overt OGIB might be expected to yield different results.

One strength of this study is that all patients with acute overt OGIB underwent CTA before enteroscopy, except for those with contraindications to CTA. This protocol could minimize selection bias; however, there were also limitations in this study. First, although it was recommended that patients with overt acute OGIB undergo CTA before enteroscopy, not all patients with positive CTA findings underwent subsequent enteroscopy. It was recommended that hemodynamically unstable patients with evidence of extravasation on CTA undergo emergency conventional angiography, and that some patients with small bowel tumors found on CTA undergo surgical treatment directly; however, in theory, the diagnostic yield of CTA would have increased if these patients had been enrolled in this study. Second, the risk of contrast nephropathy may limit the use of CTA, especially in patients with renal insufficiency. In the present study, CTA was not performed in 12 of 83 patients (14.4%) because of renal insufficiency. Therefore, these results did not necessarily apply to all patients with acute overt OGIB.

## Conclusions

CTA is useful in the diagnosis of acute overt OGIB, especially in patients with bleeding from small bowel tumors. In addition, it also can show the precise location of bleeding, and guide subsequent enteroscopic management.

## Abbreviations

CE: capsule endoscopy
CT: computed tomography
CTA: computed tomography angiography
GI: gastrointestinal
GIST: gastrointestinal stromal tumor
OGIB: obscure gastrointestinal bleeding
SBE: single-balloon enteroscopy
