# Correction: Role of computed tomography angiography on the management of overt obscure gastrointestinal bleeding

**DOI:** 10.1371/journal.pone.0193793

**Published:** 2018-02-28

**Authors:** Chao-Ming Tseng, I-Chang Lin, Chi-Yang Chang, Hsiu-Po Wang, Chih-Cheng Chen, Lein-Ray Mo, Jaw-Town Lin, Chi-Ming Tai

In the Funding section, the grant number from the funder E-Da Hospital, Taiwan is listed incorrectly. The correct grant number is: EDAHP105041.
